# Bread and Shoulders: Reversing the Downward Spiral, a Qualitative Analyses of the Effects of a Housing First-Type Program in France

**DOI:** 10.3390/ijerph15030520

**Published:** 2018-03-14

**Authors:** Pauline Rhenter, Delphine Moreau, Christian Laval, Jean Mantovani, Amandine Albisson, Guillaume Suderie, French Housing First Study Group, Mohamed Boucekine, Aurelie Tinland, Sandrine Loubière, Tim Greacen, Pascal Auquier, Vincent Girard

**Affiliations:** 1 Aix-Marseille University, Public Health Research Unit EA 3279, 13005 Marseille, France; prhenter@yahoo.fr (P.R.); lavalchristian2@gmail.com (C.L.); boucekine.m@gmail.com (M.B.); aurelie.tinland@gmail.com (A.T.); sandrine.loubiere@univ-amu.fr (S.L.); pascal.auquier@univ-amu.fr (P.A.); 2 Research Laboratory, Maison Blanche Hospital, 75018 Paris, France; delphine.m.moreau@gmail.com (D.M.); tim.greacen@ch-maison-blanche.fr (T.G.); 3 Observatory Regional of Health, Midi Pyrenees, 31000 Toulouse, France; j.mantovani@orsmip.org (J.M.); a.albisson@orsmip.org (A.A.); g.suderie@orsmip.org (G.S.); 4 French Housing First Study Group, 13000 Marseille, France; 5 Department of Research and Innovation, Support Unit for Clinical Research and Economic Evaluation, Public Hospital of Marseille (AP-HM), 13005 Marseille, France; 6 Community Mental Health Outreach Team, MARS (Movement and Action for Social Recovery), Public Hospital of Marseille (AP-HM), 13005 Marseille, France; 7 Regional Health Agency PACA (ARS), 13002 Marseille, France

**Keywords:** qualitative research, intervention research, Housing First, recovery, homelessness, trajectories

## Abstract

This paper is a qualitative analysis of the effects of *accompagnement*, a support framework, on recovery trajectories of people with long-term homelessness and severe psychiatric disorders during 24 months in a Housing First-type program in France. A comprehensive methodology based on grounded theory was used to construct an interview guide, conduct multiple interviews with 35 Housing First participants sampled for heterogeneity, and produce memos on their trajectories before and after entering the program based on interview information. Thematic analysis of a representative subsample (*n* = 13) of memos identified 12 objective factors and 6 subjective factors key to the recovery process. An in-depth re-analysis of the memos generated four recovery themes: (1) the need for secure space favorable to self-reflexivity; (2) a “honeymoon” effect; (3) the importance of even weak social ties; (4) support from and hope among peers. Three challenges to recovery were identified: (1) finding a balance between protection and risk; (2) breaking downward spirals; (3) bifurcating the trajectory. This study provides new insight into the recovery process, understood as a non-linear transformation of an experience—the relationship between objective life conditions and subjective perception of those conditions—which reinforces protective support over risk elements.

## 1. Background

### 1.1. The Housing First Approach to Homelessness and Psychiatric Disorders

In the early 1990s, Housing First-type [1] programs introduced a new supportive strategy similar to the French notion of *accompagnement* and based on the observation that the majority of people with long-term homelessness have severe psychiatric disorders [2]. Three main principles differentiate these Housing First interventions from usual housing for homeless people with mental illness: (1) the offer of a range of apartments to choose from, in accordance with neighborhood preference; (2) the choice of accepting psychiatric treatment or not; (3) a harm reduction approach to legal and illegal use of psycho-active substances. Housing First programs include intensive supports and follow-up similar to those of Assertive Community Treatment (ACT) teams and have been evaluated with randomized control trials (RCT) designs. RCT studies found the Housing First model to be more effective and efficient compared to other housing programs, in the United States [3] and Canada [4]. In fact, better effectiveness has been shown through housing stability as well as reduction in hospitalization, juridical system contact and symptoms [3,5,6].

Qualitative evaluations have also been conducted in the United States and Canada [7,8,9,10,11,12,13,14], providing a better understanding of clients’ subjective experiences when they are given access to a home after years on the streets. These qualitative studies suggest some positive effects of Housing First programs similar to those shown by epidemiologic and other quantitative studies. Such effects include: housing stability, better mental health, fewer hospitalizations and reconnection with family. The qualitative studies also allow for the description of more complex, subjective effects, such as a feeling of security and pride in having a home [10]; the sense that the moment is right for reconstructing one’s biography, identity and self-presentation to others [10,11,12,13]; and positive socialization and a sense of hope [7,8,14].

### 1.2. Recovery, Homelessness, and Psychiatric Disorders

The subjective experiences described in the Housing First qualitative studies are often examined through the paradigm of recovery. Today, this concept, co-constructed by service users and researchers in North America beginning in the late 1980s [15], structures public mental health policy in several countries and influences professional practices [16].

Although the everyday meaning of recovery implies a return to a premorbid state or the recuperation of something that has been lost, the recovery paradigm in mental health implies a particular, non-linear process without an emphasis on an endpoint. This paradigm evolved from knowledge gleaned through patient narratives [17] and from the results of longitudinal studies of severe psychotic disorders. Rather than assuming deterioration or immutability, patients’ recovery experiences have focused on resilience, transformation through the discovery and acceptance of one’s capacities and limitations, finding meaning and a degree of autonomy in life, and empowerment. That change is possible even for people with severe psychiatric disorders was suggested by the considerable percentage of patients who returned to work and/or the community, in cohorts followed by Strauss and Carpenter, Harding and others [15]. These two strands—experiential and epidemiological—have influenced the development of patient-centered, recovery-based psychiatric rehabilitation [18] and have found their way into various European, national- and (in the U.S.) state-level recommendations and policies [19,20].

The recovery paradigm and its evolution over the past thirty years have been reviewed by Amering and Schmolke [20]. They identify three major components of recovery: an active process of gaining security and control over one’s life; an orientation towards “becoming whole” or integrating the awareness of one’s illness or condition into one’s life; and the recognition of improvement and change, without denying one’s symptoms and condition. Further classify the components of recovery into internal (hope and health, or the sense of self-worth beyond illness) and external (vocational and social reintegration). Other researchers emphasize the competencies necessary for engaging in a process of recovery and a larger sense of reconnection to others [21].

In a more political framework, recovery includes both the above individual-level components and a collective dimension, such as the public expression of the ability to overcome one’s mental illness and its social and political consequences [22].

Housing First programs are considered by many actors to be a recovery-oriented approach [17]. This is true of the multi-site, RCT-designed Canadian intervention, At-Home/*Chez soi*. An important qualitative component examined the program’s effects on the recovery of participants over an 18-month period. Early results suggested that having a home provides a necessary basis for imagining the future and developing a reflexive self [13]. A second study described how the program generates hope. A third series of results [10] identified four changes from the participant’s point of view: (1) a feeling of security and pride linked to the experience of having a home; (2) challenges—especially those related to isolation and personal identity—associated with adapting to a new environment; (3) having a home as a learning process that develops over time; and (4) the significance of meaningful daily activity as opposed to mere survival. This article also defined four factors which either facilitated or obstructed changes: (1) the availability and quality of services and support; (2) the effects of trauma; (3) the availability of social support; and (4) current obstacles to the use of psycho-active substances. Finally, a last article [23] identified three trajectories of positive transition linked to residential stability in the process of recovery: from street to home, from home to community and the development of the ability to project a future-orientation in the present. One conclusion highlights the role of material conditions of existence, such as housing, in the expression of a sane self, and the relationship between structural conditions and the process of recovery.

### 1.3. The Recovery Paradigm in France

Although Canadian researchers accept the need to foster the relationship between access to a home and recovery among people living with psychiatric disorders [24], this orientation has been far from obvious in the French context, including for the Housing First research team. A major reason is that the recovery paradigm was until recently unknown in France. French peer-worker programs experimented with the notion in the late 1990s [25,26]. Around the time that psychiatric disability (*handicap psychique*) was first defined as a legitimate type of disability, in a 2005 French law [27], a small number of French researchers were calling to de-emphasize a solely medical perspective in favor of fostering recovery. They defined recovery as a dimension of psychosocial rehabilitation in which patients themselves were agents [25,26,28,29].

Despite these first steps, no two teams in France have used the recovery concept in the same way today, with rare exceptions [30]. The French Housing First program (*Un Chez Soi d’Abord*), from which the research reported here is drawn, has been central to importing, diffusing and further developing the recovery concept in France [31]. An earlier study nevertheless examined how a recovery-orientation, defined as patient-centered goal-setting and choice, comes into conflict with professional care in French Housing First programs [26]. However, what constitutes recovery from the patient’s point of view and what recovery trajectories look like within the French context have never been examined. The present study aimed to explore this question within the *Un Chez Soi d’Abord* program.

## 2. The French *Un Chez Soi d’Abord* Qualitative Study: Overview

### 2.1. Presentation of the Study

The present study, a qualitative component of a randomized control study entitled “The course of recovery in the *Un Chez Soi d’Abord* program in France” [32], aimed to identify elements that constitute recovery within the specific context and research culture of French professionals and stakeholders. A comprehensive qualitative research design was developed to explore support and recovery among persons with severe psychiatric disorders and long-term homelessness, with a focus on the patients’ experiences. The methodology differs somewhat from that of the Canadian qualitative studies cited above. While the Canadian researchers built their hypotheses from scientific literature reviews, the present study privileged hypotheses developed from field experience. This inductive approach, well-known to qualitative researchers yet rarely used as a complement to RCTs, is based on “grounded theory” [33]. It involves a qualitative process of identifying notions used by the social actors themselves rather than imposing external concepts on their actions and behaviors. Furthermore, it uses a recursive process of data gathering (through observation and interviews), analysis, and theory building. Hypotheses that emerge from fieldwork are tested and refined through further data gathering. This back-and-forth movement between field and analysis produces constant modifications in the theories and concepts.

### 2.2. Study Context:The Un Chez Soi d’Abord Randomized Controlled Trial Study

The qualitative study was developed to complement the French *Un Chez Soi d’Abord* program, a multi-center randomized controlled trial (RCT) study conducted in four large French cities (Lille, Marseille, Paris, and Toulouse). The qualitative sample was derived from the overall RCT sample (*n* = 703).

Inclusion criteria for the larger RCT study were as follows: over 18 years of age; absolute homelessness (i.e., no fixed place to stay for at least the past seven nights with little likelihood of finding a place in the upcoming month) OR precarious housing situation (housed in single-room occupancy, rooming house, or hotel/motel as primary residence AND a history of two or more episodes of absolute homelessness in the past year OR one episode of absolute homelessness lasting at least 4 weeks in the past year); diagnosis of schizophrenia (SZ) or bipolar disorder (BD) made by a psychiatrist according to Diagnostic and Statistical Manual of Mental Disorders, 4th Edition (DSM-IV-TR) criteria [34]; and the ability to speak French. Exclusion criteria were: reduced capacity for consent [35,36]; having young or dependent children at the time of the evaluation; currently pregnant; and/or irregular immigration status in accordance with the French law on biomedical research.

Mobile mental health outreach teams [37] in each city recruited RCT study participants from August 2011 to April 2014, from the street, emergency shelters, hospitals, and jails. Once they were included in the study, those randomized to the experimental group received housing immediately. The RCT methodology has been described elsewhere [32].

The study was conducted in accordance with the principles of the Declaration of Helsinki, 6th revision. All participants provided written consent. The local ethics committee (Comité de Protection des Personnes Sud-Méditerranée V, France: trial number 11.050) and the French Drug and Device Regulation Agency (trial number 2011-A00668-33) approved the study.

### 2.3. The Qualitative Study Component

**Figure 1:**
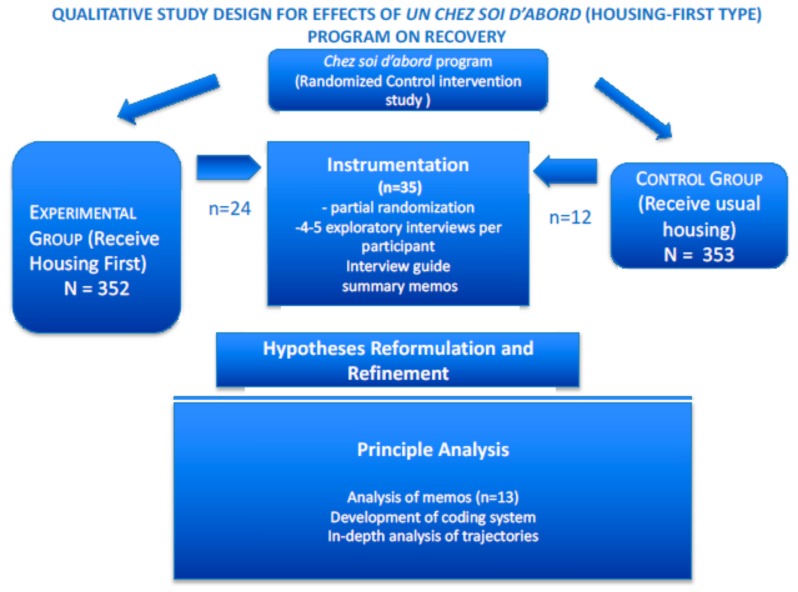
Research Design of Qualitative Study.

The qualitative study reported in this paper took place in three overlapping phases (see Figure 1). During the *Instrumentation Phase* (2012), one research sociologist in each city conducted four or five interviews with each of 35 participants qualitative study participants (sampling is described below). These initial open-ended interviews aimed to delineate the domain of housing and recovery experience. After an analysis of the set of initial interviews, an interview grid was developed and used for the remaining two to five interviews per participant. Researchers then developed a memo summarizing material in all interviews of each participant, for a subset of the qualitative study participants.

During the *Hypothesis Reformulation* phase (2013), the research object was reformulated on the basis of interview content. In particular, the subjective and objective conditions of recovery were identified.

During the last, *Principal Analysis* phase, several types of data analysis were undertaken by researchers from two sites, a sociologist and a public health researcher/psychiatrist. Recurrent themes were extracted from a subset of 13 memos (from the Instrumentation Phase) and a coding system of progression along factors of recovery was developed. The memos were again re-examined, with these factors in mind, to develop typology of trajectories.

### 2.4. Sampling Procedures

The qualitative sample was selected from the RCT overall sample (*n* = 703) in four cities and hence met inclusion and exclusion criteria of the RCT study (see Table 1). Thirty-six participants were recruited for the qualitative study from the experimental group (*n* = 24) and the control group (*n* = 12), using random selection when possible and respecting the predetermined RCT breakdown of 70% (experimental) and 30% (control group). Randomization was halted to increase the recruitment of women (every nth female rather than every nth participant). Fifteen percent (*n* = 5) of the final sample were women. All participants provided consent for participating in the qualitative study and chose where to be interviewed. The total inclusion period was 8 months, and data were collected over 36 months, from September 2011 to September 2014.

The final sample of 35 (one participant had died) included 10 individuals from each of two cities, 9 from the third city, and 5 from the fourth city (which was limited by staffing problems). All but one experimental group participant was housed by the time the interviews began.

### 2.5. Analytic Strategy

The qualitative research team (three research sociologists and one research sociologist/coordinator) opted for a methodological approach based on Grounded Theory. This choice involved using exploratory non-structured interviewing, participatory observation and informal conversations, informal conversation and non-directive interviews, but with only preliminary ideas about what constituted the domain of recovery from the participants’ viewpoint. Data were elaborated and revised into firmer hypotheses as the study evolved.

In addition to exploring notions of recovery particular to French culture, the choice of an inductive approach was motivated by the fact that a model of *accompagnement* (support) influenced by Assertive Community Treatment (ACT) but always including peer workers had yet to be tested in France. Thus, exploratory research was needed to describe the practices used in the French program. The innovative nature of the *Un Chez Soi d’Abord* program also led researchers to hypothesize that the content of the practice of *accompagnement* would both evolve over time and impact recovery trajectories in different ways. To meet this initial challenge, researchers sought specific information on how the *accompagnement* team had been recruited, how participants accessed housing and were accepted by different local program contexts, how electoral politics and national policy affected trajectories, and so forth. Grounded Theory seemed particularly suitable because it captured the dynamics of trajectories and contextual conditions while allowing for the back-and-forth movement between data collection and analysis and the refinement of variables and hypotheses over time [38].

Finally, from the start, the researchers privileged the voices of the users, considering that the discourse of people with even severe psychiatric disorders is at once pertinent and unique [39,40,41], as long as research mechanisms provide the means for capturing those voices. Furthermore, allowing voices to emerge was essential to the research perspective itself, as recovery involves, by definition, a personal process, without third-person interpretation. This led to further formulations of key concepts, as we shall see.

In the rest of this paper, we detail the three phases of the qualitative study:

Instrumentation, Hypothesis Reformulation, and Principle Analyses, as illustrated in Figure 1. The Instrumentation phase describes how the interview guidelines were developed, including early data collection, preliminary analyses, and summation. The Hypothesis Reformulation section describes how the factors of recovery were identified in terms of the early data. This reformulation rejected the notion of recovery as a monolithic positive phenomenon. The Principal Analysis section which includes the most in-depth analysis of this qualitative study, describes the development of a scale from the data already collected in the first phase. The scale enables recovery to be measured as a progression along two dimensions, Objective Factors and Subjective Factors of recovery. This scale was further applied to a subset (*n* = 13) of the memos from the first phase and a three-fold typology of recovery trajectories was extracted. The results of this in-depth re-analysis of the data are illustrated with cases based on the study participants’ own words. The Discussion section presents recovery *à la française* as it emerges in the study results and proposes a re-conceptualization of recovery more appropriate to French research and clinical culture.

## 3. Instrumentation Phase and Data Collection

### 3.1. Early Explorations and Instrument Development

After the original 36 participants were recruited, the researcher sociologist at each site was instructed to identify events that tended to re-occur in their lives. As both participants in the experimental and the control group had long-term homelessness and severe psychiatric disorders, such events in the past were assumed to be unaffected by whether they were assigned to *Un Chez Soi d’Abord* or not. These events included: reduction in substance use, re-establishment of the parent role, obtaining a job, avoidance of boredom, entry into treatment.

Each participant was initially interviewed one or two times. The interview was open-ended and recorded. These initial interviews sought to circumscribe the domain to be explored, along the following dimensions: challenges, constraints, and favorable circumstances through which participants withstand their life conditions, relationship to their body and their health, aspirations and projections for the future, relationship to home, past and present relationships to services offered, social relations (friends, family, urban acquaintances), places for socio-cultural activity, leisure, and pleasure; sense of security/insecurity, social utility (useful, useless), well-being (absence, presence), awareness of the world surrounding them; beliefs, spiritual references, and relationship to the religious (see Table 1).

Several practical issues were also explored over the first year, such as how to adapt observation and interview modalities to a longitudinal dimension, counter the narrative vulnerability of participants used to presenting their biography in conformity with the expectations of social workers, handle the effect of intermittent psychiatric symptoms on the interview and treat the participants’ lack of knowledge about the recovery notion. This exploratory phase of data collection also revealed the great disparity among participant’s personal situations and profiles, which the qualitative methods had to be sensitive enough to capture. Finally, during this period, researchers documented early perceptions of the control group. In particular, the effects of their participation in a research program led some to embark on a path of recovery.

### 3.2. Formalization of Interview Guidelines and Semi-Structured Interviews

Delineation of the interview domain and results from these first interviews allowed the researchers to elaborate a more formal set of interview guidelines at the end of the first year, to be used in the subsequent 2 to 4 interviews (see Table 2). The new guidelines allowed the researcher to explore the evolution of personal dynamics in the participant’s experience after entry into the program (experimental or control group). Such questions included: who or what is helpful? What possible choices, alternative existence, effective choices, non-choices does he/she envision? What challenges are involved in the reconfiguration of identity? What types of learning result through experience? What are the challenges to appropriating the power to act? The practical challenges of citizenship?

Overall, researchers conducted between four and five interviews per person, using the formal guide, taking an average of 23.07 months per participant. All initial open-ended and subsequent semi-directive interviews were recorded and transcribed, with the exception of those conducted in prison. They lasted 55 min on average.

In addition to interviews, informal conversations took place in various contexts: homes, group gatherings whether organized by the same team or not, shelters, hotels, hospitals, rehabilitation centers, prison, participant meetings, local institutional meetings, national awareness and dialogue events organized by program coordinators. Information from these sources was added to the interviews when relevant.

## 4. Hypothesis Reformulation

Preliminary analyses of the interviews were carried out by the research team in periodical meetings (about every 6 months). These led the research team to reformulate the research objectives by elaborating the notion of “subjective experiences of recovery” and mobilizing a pertinent body of literature centred on the concepts of capability [42], challenge [43], protective support [44], and bonding and bridging [45]. This phase did away with the initial diametrically opposed conceptual pairs—such as recovery/renunciation, dependency/freedom, and normalcy/marginality—now found to be incongruent with the actual experience of participants. The team also decided to deepen the question of citizenship [46] through a series of individual interviews at each site, and to explore the way in which program participants developed collectively and through public voices.

After all interviews were completed, data collected in the four cities were pooled. The qualitative research team developed a common grid to organize the interview material, based on four themes: housing, psychiatric disorders, addictions, and relationships. Researchers also composed a series of memos that summarized the experience of each participant as indicated in their interviews. Four broad domains areas were covered: (1) sociodemographic characteristics and history: age, family, salient events in the life course, illnesses and health, experience on the streets, residential trajectory; (2) abilities, challenges; (3) the program’s place within the person’s personal development (for the experimental group participants); (4) more general information concerning the recovery process.

This preliminary phase of the study confirmed the irreducibility of different recovery experiences to a list of objective elements of “good recovery”, leading to the rejection of such a list. Analysis of the interviews further led to an understanding of the recovery process in terms of the dynamic relationship between interior and exterior forces [47], the passage from the access to goods (housing, money, other) to the actualization of the capacity for action [20] and adaptation to daily events [21]. The hypothesis developed at that time is that, for each person, the recovery process builds on different interactions over time between objective and subjective elements.

These preliminary conclusions led the research team to consider subjective experiences of recovery by way of a new analytic grid according to whether elements involved risk or protection, internal or external factors, and were linked or not to the program.

## 5. Principal Analysis: Typology and Results

### 5.1. Analytic Strategy

The final stage of this study produced the principal analyses to date of the qualitative component of the *Un Chez Soi d’Abord* program. For this analysis, a coding scheme and scale were developed from a systematic examination of the memos selected from Phase 1. The scale and types were then applied in an in-depth analysis of the trajectories of 13 of the original 35 research participants. These analyses are presented in the next section.

A sociologist and a public health researcher/psychiatrist from two different *Un Chez Soi d’Abord* sites re-examined some of the materials collected in the Instrumentation Phase. They developed a panel of 13 (out of 35) trajectories: 9 from the experimental and 4 from the control group. Sampling for the panel followed the principle of heterogeneity, to ensure a diversity of biographical pathways. Men were overrepresented (12 of the 13 participants); age varied from 23 to 60; duration of life in the street from a few days to 12 years; health states from good physical health to several long-term illnesses; alcohol and drug use from none or intermittent to permanent. Each memo, 8 to 10 pages in length, included biographical information, the list of interviews made, issues or major challenges faced, and citations from the interviews. All information in the memos was extracted from interviews with the participants.

To further identify what affects recovery, the two researchers independently re-examined these memos using thematic analysis to identify recurring themes related to objective life conditions and to participants’ subjective perceptions since their integration into the program. The researchers then compared their results, discussed their disagreements, and reached a consensus. This operation led to the selection of twelve objective variables (OV) and six subjective variables (SV) affecting the trajectories (see Table S1 in the supplementary material). The twelve objective variables were: residential stability, establishing rights, social ties, family ties, couple relationships and sexuality, leisure activities, somatic health, mental health, access to medical care and to alternative resources. The six subjective variables were: hope/projection into the future/goal development, reflexivity, identity and social roles, sense of security, control of symptoms or the disorders, elaboration of strategies.

Memos were examined a second time, again by each of the two researchers separately, to elaborate a coding system. Developments observed for each of the 13 participants between the first and last interview were given a value of 1 to 5. The coding system retrospectively integrated participants’ situations before entering the program.

Scaling allowed the calculation of an “objective variable” score and a “subjective variable” score for each trajectory memo. Individual scores were determined for each of the eighteen factors, based on a scale of 1 to 5. The twelve Objective Variable scores were then summed and averaged to provide a Summary Calculation, on a scale of 0 to 60. The six Subjective Variables were similarly scored to provide a Summary Calculation on a scale of 0 to 30. For each participant, it was possible to further calculate what percentage of the highest possible Objective and highest possible Subjective score his/her actual scores represented. The results for each participant are presented in Table S2, in the supplementary material. These scores were then used to test the hypothesis of a relationship between the development of objective and subjective variables.

The final analysis of the 13 memos produced a three-fold typology: (1) Downward trajectory with cushioning effects: the downward trajectory is present at entry into the program, and the program’s effects are either neutral or insufficient on the trajectory itself; (2) Irregular or “jagged” trajectory with intermittent positive effects: the trajectory alternating periods of hope and renunciation suggesting intermittent program effects; (3) Split trajectory, with a rebound effect: the path towards recovery bifurcates, showing positive effects of program.

The two researchers classified the 13 trajectories separately and by type, then compared their respective classifications, sharing interpretations and discussing the elements of consensus and non-consensus. Consensus was found for 11 trajectories and no consensus for 2 trajectories. The Summary score for each participant on the Objective and Subjective Criteria of Recovery Scale are presented in Table S2 in the supplementary material.

The next section presents the themes that emerged from these three types of trajectories.

### 5.2. Themes Emerging in Recovery Trajectories

In-depth analysis of participant comments in the memos generated fours themes.

#### 5.2.1. A secure Space Favorable to Self-Reflexivity: “Settle down First, Start Thinking Later”

Participants first express the need for shelter and sometimes for distance from the tough times and/or violence of life on the streets. These experiences, often marked by violent and traumatic events, are recurrent and lead people to modify or to distance themselves from social relationships associated with wandering. Being able to settle down and be relaxed in a home reenacts the memory of street experiences or previous psychiatric journeys in which ontological security disappeared. For many, being sedentary mainly means protecting oneself against street violence and exiting a survival mode (“I would roam the streets at night, and sleep in parks, parking lots, or benches during the day”, “I ate from garbage cans”) or escaping the constraints of shelter life (“I had to stay outside during the day”). Study participants provided personal criteria of what constituted good and bad shelters, good and bad psychiatric services. Generally, however, early on in the trajectory, the viewpoint took the form of complaints about institutions (mainly psychiatry and social work) in which the figure of the victim or the dependent or disabled person resurfaces.

Once housed and followed by the *Un Chez Soi d’Abord* program, participants tended to present their experience in terms of before and after. Finding housing again involves a first step towards the reconstruction of one’s identity [13].

Within personal journeys, the bifurcation that materializes in being re-housed raises questions once again about their previous life. For many, housing, having slowly become something in which they can invest themselves, forces a reflection on past and present behaviors. The first perceptions of a new situation are all the more marked if the past was experienced as very painful (violence, long-term wandering).

In terms of perceived health, participants reformulate their relationship to disorders, anxiety, addictions or inner voices. In the words of one participant: “What is important to me is to feel secure, weaken the impact of my inner voices on my life, and limit the side effects of addiction and prescription drugs”.

A reflexive self-interrogation including health leads to the examination of “destructive” behaviors of how to protect oneself from feelings or violent acts (“My old life, it was another life”) and choose one’s relationships. It increases the range of possibilities from which to choose and allows one to formulate new goals [8].

#### 5.2.2. The Program’s “Honeymoon Effect”

Most participants experienced the early days of the *Un Chez Soi d’Abord* program like a true honeymoon, marked by deep gratitude towards the team of professionals. The following citations from five different people shortly after moving into housing express this phenomenon:

*“Before the program, no one helped me, I was hustling on my own.”*

*“It’s the first time I have had a choice. With this apartment… it’s the end of tough times.”*

*“Without Un Chez Soi d’Abord program, I never would have gotten an apartment.* *”*

*“I sleep better, eat better, I feel secure… The team brings me comfort.”*

*“Everything is better now. When I have a problem, I have someone I can count on.”*

Nevertheless, in terms of social trajectory, access to the experimental program has heterogeneous effects depending on age, sex, and length and type of street experience (from violence to the experience of some form of solidarity). To have a home is not only to have a mailing address, but also to personalize the space one lives in (furnishing, cleaning, decorating). But being housed also means new worries: paying rent, feeling lonely, avoiding having squatters, repaying past debts, being locatable by the justice system, and continuing to fight one’s voices. The “honeymoon” ends by ceding to alternating periods of hope and renunciation.

The “honeymoon effect” is illustrated from interviews taken shortly after intake into the program, at three months, and at twelve months:

T 0:
My life is already better. I never thought that one day I’d have my own place. I had tears in my eyes when I got the envelope … I’m going to go to the self-help group, I’ll do everything I can to go… I hope I can hold myself together until my daughter has a child. I hope I’m not going to do what I used to do, wanting to end my life… Before, my life had no meaning.
T + 3 months:
No one loves me for who I am [he cries]. For years, I kept everything to myself, and now I’m just exploding and I turn away all the people who try to understand me.
T + 12 months:
I don’t like the Un Chez Ssoi d’Abord program’s group outings…First of all, I don’t want to see them. I hardly ever see [my partner]. Her place is tiny. She’s sick… It’s my weight that’s a problem. I lock myself in my house. I don’t even own clothes anymore… I went off my treatment cold turkey, it’s been six months, I know I got fat because of that… I don’t want to see a doctor… I no longer hear from my two children… Nothing is interesting to me except movies… I’m behind on my rent. I spent everything betting on the horses.

#### 5.2.3. The Importance of Social Ties, Even When Weak

The concern with getting one’s life together depends in varying ways on the *accompagnement* or ongoing support proposed by the team’s professionals. While certain people say they can and should always rely on themselves, others perceive the *Un Chez Soi d’Abord* team as at least a security blanket and at most a generator of hope. The framing of the interviews offers participants the possibility of reflecting back on the past, which in turn reveals how sensitive the interpretation of the development of their trajectory can be. The individual in recovery is generally presumed to develop relationships based on the model of social inclusion. Recovery becomes a synonym for the intensification of informal and formal ties among peer, proximal ties with non-peers and less asymmetrical relationships with social and health workers. But recovery also implies a break with past relationships. For example, as soon as they join the study’s experimental group, some people completely break away from the culture of past street life: relationships with peers, neighborhood shopkeepers and members of non-profit organizations. Another participant chose to live in the suburbs but for months kept returning to the streets downtown, where he would live under the benevolent gaze of shopkeepers in the very neighborhood where he used to beg. At a later point, he kept to himself and developed “exclusive” relationships with team members and health professionals. Finally, the nature of these ties varies according to the level of constraint they impose. Finding a balance can also involve exposing oneself less often to constraining relationships. For instance, one participant sees other people more often than two years ago, but doesn’t necessarily have any friends (“*I am a sociable loner*”). He knows many professionals (the *Un Chez Soi d’Abord* team, his general practitioner, a private nurse), patients from the day hospital and rehab center, other *Un Chez Soi d’Abord* program participants. He experiences his relationships similarly to the positive retreat described by Ellen Corin [40]. This modality involves being with people without having to interact with them, or without doing so in an explicit way. In other words, forming social ties, even when they are weak, counts. Frequenting particular places is both solitary and inclusive, whereas being amongst others does not systematically bind an interaction. Social ties, even when weak, are at the same time strong. Many participants share a willingness to defer rebuilding social relationships until their life becomes more stable [48].

Weak ties are therefore low-dependency ties: the individual is less exposed. On the contrary, strong ties expose the person more often to risk but are also a more effective source of support in difficult situations. The choice of one’s social relations as a marker for recovery has been largely described in the Housing First literature, but also beyond, whether it be in termos of reconstructing relationships with “trusted others” [21], elaborating a routine with choosing one’s social relationships [23] and developing or rebuilding “positive relationships” [8].

The future of family ties in the *Un Chez Soi d’Abord* program involves the same mechanisms. First, the process of actively rekindling family ties undeniably marks a step in the recovery process. The following 38-year-old man got in touch with his daughter and parents immediately after being re-housed:

*“My daughter… when I was homeless… I totally lost her. To find my father, I had to go to the cops, you know, like, start an investigation. And you know, he had really helped me when I was a kid. And then I’ve got memories—I can’t forget them. So I wanted to get in touch again. And then this weekend, he set up a meeting. He called me and he said, “I want to see you again”. So once in a while we’ll see each other. I took the train there yesterday. He was welcoming. Yeah, I was pleased.”*

However, distancing oneself from one’s family does not necessarily imply “non-recovery” and must be interpreted on a case-by-case basis. One person might still be embarrassed by their situation, another feels that reconnecting with their family might upset a barely achieved state of tranquility, and still another lucidly identifies their family as structurally harmful:

*“It’s ancient history, I feel relaxed now. It’s been too long now. It’s been seven years. I know they know I was homeless. If they don’t know, that’s for the best. I would be ashamed because they’ve been working for several years; they have children, they have their house. Me, I didn’t do anything for 6 or 7 years. My mother, I know where she is. But it’s not good to talk to someone who’s bipolar, and the other way around, given her mental state.”*

*“* *My family? Oh, no, they’re miserable, always problems, fights, misunderstandings. It tires me out just talking about it. …my mother fell into alcoholism; my father is unhappy because his children didn’t do the right things like he wanted… My 22-year-old younger brother still rules at my mother’s house, and it’s difficult, so I’m in a hurry to leave… I’m leaving Friday and that’s already too long and I’m happy to leave. If not, there will be a murder. If I don’t leave, there will be a murder.”*

#### 5.2.4. Hope and Peer Support Give Meaning to Life

The first conclusion stemming from the principal analysis is that no unequivocal link exists between the development of objective and subjective variables, and this is true for both groups. In other words, the recovery process observed in this study does not necessarily designate an improvement in objective conditions but rather in the subjective definition of improvement.

Within the *Un Chez Soi d’Abord* program, the *accompagnement* or support that accompanies accessing housing is sufficient for basically transforming the participant’s objective life conditions, in terms of social status and resources such as health states. But in general, the subjective relationship between participants and their objective conditions has changed, even when improvement is slow. For example, we observe that the forms through which experience is expressed, and its tonality, have often evolved.

For example, a 30-year-old man who had worked on making his home into a place of refuge and sustenance by contrast with his traumatizing experience of anonymous and violent street life, ended up in a very difficult financial situation two years into the program because he had used all of his disability entitlements to pay off a debt to his dealer. He could not pay his rent, which was unusual for him. Yet this period corresponded to a moment in which he becoming capable of taking control of his life by projecting a future life. He asked to be placed under guardianship, realizing that his addictions were jeopardizing his ability to keep his living space. He had been abstinent for a month, and his inner voices attenuated after a peer-worker from the Hearing Voices group provided support. Those states clearly allowed him to improve his life despite going through a period of objective deadlock. And although when he first entered the program, his self-reflexivity was weak and he could not talk about his experience other than in the terms of a routine, reassuring and non-adversarial everyday life; he now began, for the first time, to use terms referring to pleasure (“pleasant”, “nice moment”, “makes me glad”), hope (“*Now I only have one voice, it’s a big victory*”), self-esteem (“*I am a friendly person*”). In addition, the tone of his rhetoric changed as he laughed and joked about his situation, no matter how fragile it was. The vocabulary with which he described his experience changed (“*Well I’m in hot shit now*”), as did its style (humour and self-derision), even though he was still struggling with the nagging problem of his illicit addictions.

However, the feeling of greater well-being, hope, and a projected future are not limited to the members of the experimental group. Control group participants also experienced positive biographical conversions. For members of both groups, the recovery process is related to the participant’s being able to rely on support from his or her entourage and the becoming involved in a meaningful job or social activity. An undeniable link exists between regaining self-esteem and changes in how their life and health conditions are viewed by significant others. Some participants contend that participating in research, even in the control group, helps them understand the obstacles they face (“*I’d like to read my interviews to understand/check in on my life*” “*I am not displeased with the fact that people are interested in my experience*”).

Therefore, if obstacles (e.g., limited monetary resources, precarious living situations, deficits in social support…) to the satisfaction of basic needs accumulate alongside stigma and denigration by others, it becomes difficult to create an experience of recovery, particularly hope. This observation resonates with Patterson et al.’s analyses [10], which pinpoint distrust of the system and conditional access to housing as negative factors in the recovery process. An example is a 60-year-old man in the control group, who spent months in a shelter waiting for his retirement pension to be processed after his disability entitlements were cut off. The shelter where he was stranded turned out to be a turbulent environment that upset rather than soothed him. His situation put him in a deadlock, and he had no one to count on:

*“We are not free in the shelter. At my age, freedom is important. I can live in a community if that community respects certain rules. It’s not easy. Sure, I need structure, but with structure there are always rules. If a person does not respect the rules, it creates bad vibes. The [shelter* *’s] schedule is one thing—* *waiting in the streets for three hours. I would go to M [another shelter], but you know, there* *’s a ton of people there and I don’t feel comfortable. It* *’s full of lost souls. How can I pull through if I hang out with them? Right now, I don’t have the energy. I need to socialize with people who aren* *’t losers.* *”*

By contrast, the *Un Chez Soi d’Abord* team, which attempts to provide hope and takes care not to reproduce the usual stigma, often takes on the role of a supportive entourage. This is clear in the words of a young man who at first didn’t dare discuss his mental breakdown with the team, but eventually changed his mind after being hospitalized:

*“Honestly, that’s the shitty mistake I made. If I had to talk to people who did not feel comfortable with themselves. Asking for help is not a weakness, maybe it’s even a strength. When we ask for help and we receive it, it means that on the one hand, there’s someone there for us, and that’s a strength not a weakness… it’s better to have someone around. What happened to me, is that when I started freaking out, being paranoid… I tried to fight it alone. I wouldn’t dare to… I had a feeling—how can I put it—of embarrassment. And I would tell myself that I’ve been in top shape for the past two years: I have an apartment, I’m self-confident, I was able to finish my studies and then all of the sudden I start going downhill, just like that. I didn’t want people to know. But the best thing to do is still to talk about it. The team [assigned to me], they are important. As much as they ever were, except it* *’s my turn to ask for their help. They are still as important, but if I don’t go see them, they won’t come to me. They aren’t in my head”.*

### 5.3. The Complexity of Recovery Trajectories

#### 5.3.1. A Balance between Risk and Protection

The retrospective analysis of life trajectories through memos allows us to understand that a same phenomenon may be qualified as both an element of risk or an element of protection, depending on the individual’s perception and on the specific period of his or her life. For example, for some participants, housing obtained through *Un Chez Ssoi d’Abord* might constitute a major element of protection against earlier experiences of street violence and social relationships linked to substance use. For other participants, housing will present an element of risk in terms of social isolation because they do not have a history of traumatic street life. Community ties are not necessarily a positive factor in terms of improved quality of life in the early stages of joining a program [49].

An increase in resources might arouse enough pride in one person for him to want to contact his teenage daughter and be able to give her small gifts. This becomes a protective factor. Financial solvency in another case drew the attention of past creditors, becoming an element of risk. A participant might experience the program’s workers as an “*affectionate presence*” “*without the ‘I told you so’ part*”, and thus as an element of protection. Yet the stigmatization of usual psychiatric services might still affect another participant such that their hospitalization presents an element of risk.

Davidson and his team describe personal recovery as a multidimensional learning process, very similar to what people with a history of street life tend to convey once they are being supported by the *Un Chez Soi d’Abord* program [21]. The factors contributing to recovery identified by these authors include material resources linked to obtaining a safe living space, with privacy and security, and sufficient financial funds to engage in meaningful activities and social relations with significant others. The present study also observed many ambivalent situations in the recovery process. Work can be stressful, subservient, and poorly compensated; housing might be not private or safe enough, social relations too stressful or disability benefits experienced as degrading. In these cases, the benefits of social inclusion are often negated. For example, Mouloud, a young man in the control group, experimented with multiple types of care, housing, and social work mechanisms. But he experienced them as ineffective and out of synchronization with his own temporality. This young man had managed to keep his housing during his ten months of incarceration. When released from prison, he was under the care of numerous professionals (social, medical, addiction specialists, guardianship). Yet he still had trouble getting enough to eat:

*“Boxes of Risperdal are all I have in my fridge. Because I was in prison, my disability check was cut by 30%. So, I only get 250 euro (instead of almost 800). I have to give social services the paper that says I was released from prison, and they’ll get me back my disability check. My guardian had to pay the landlord and the electricity bill. So, I have nothing to eat and nothing to smoke. I keep trying to apply for benefits, but as I’m on disability benefits already and I* *’m under guardianship, I get nothing.* *”*

This analysis suggests that the recovery process is a search for a balance between risk and protective elements [21]. It systematically relates a person’s situation (having a home/being homeless, having resources or not, having social relations or not) to their experience of this situation.

#### 5.3.2. Combining Factors in Recovery: Dilemmas and Vicious Circles

The complexity of individual recovery processes requires us to collectively think through data concerning experience in order to understand certain relationships. Typical examples include the relationship between a circle of friends and drug consumption, between financial security and being able to renew one’s role as a parent, between obtaining housing and solitude. The following situation is particularly illustrative of this observation.

Yann is 42 years old. He has two daughters from a previous marriage, aged 12 and 14. They live far from him, with their mother. He hasn’t seen them in several years but keeps in touch episodically by phone. His relationship with their mother is poor and essentially mediated by the judge. His housing situation has quickly improved, even though he is in the control group. After staying in a shelter, then a halfway house, he moved in with his current partner in the suburbs. His financial resources have increased, so he must pay child support arrears. But not being able to see his daughters makes him feel bitter. This situation, along with his cocaine use (and the additional guilt he feels about not being able to repay his debts), largely hinder his chances of once again becoming an effective father figure:

*“Actually, it’s been ages since we’ve seen each other (…) and I don’t have enough money to take the two trains to go visit them… Last month, my daughter asked me to give her some pocket money for the annual sales. I wasn’t able to. I stopped paying child support because I was sick of it. I did something else with that money—I bought some boots and a pair of jeans. I get no news from my daughters. They never call me… As for the child support people, they’re going to summon me soon. That bothers me a lot. I have to ask for an evaluation all over again. I feel all over the place about that. It’s pretty intense. I feel guilty for not paying child support for the past two months. I’m afraid to call my daughters because their mother might answer. I’m waiting to get a job so that I can start paying child support again (…) I could call my old lawyer (…) but I don’t have the energy. In the present situation, I’m running away from things instead of facing them. It’s my fault because I spent all my money on cocaine. If I admit that, it will be used against me. Even today, with a home, I can’t make it mentally. It’s crazy.”*

Yann used a large number of drugs between ages 18 to 30, in more or less recreational settings (“*It was the rock and roll days*”, he would later say), especially with friends who could give him a place to stay. After finding housing, he chose abstinence because the effects of the drugs had become unbearable, and incompatible with his other disorders and treatments, as well as with the financial stability he was striving to attain. He now deploys several strategies to occupy his time and is getting used to being alone—he reads, listens to music, goes to exhibits—so he can resist being won over by the addictions of others. But despite these changes, Yann does not always “*feel strong enough avoid falling back into drugs*”. His example, far from being exceptional, attests to the difficulty of keeping off drugs while maintaining a circle of friends.

This type of situation illustrates to what extent supposedly positive elements may become obstacles to the recovery process and to what extent the latter can be fragile. The search for social relationships becomes an obstacle to lowering drug consumption, and sobriety is synonymous with boredom. Improving one’s administrative and financial situation becomes a passport to debt or failure to rekindle family ties, and securing housing a source of loneliness. Thus, the interpretation of positive factors of recovery is fraught with ambivalence, as they interact and in every case depend on their place within a longer personal history.

#### 5.3.3. A Typology of Trajectories

Several observations can be made about the classification of the thirteen trajectories in principal analysis. The first take-away is that participants enter a program from heterogeneous situations. Their life experiences are diverse, as is their sense of control over their existence. Nevertheless, several points can be drawn from the retrospective analysis of the trajectories. Program participants arrive from highly variable experiences, given their life histories. The program affects their situations in different ways (see Table 2). (1) It can set off a *downward trajectory*, with a cushioning effect. For the experimental group, the effects of the program intervention are thus considerable but insufficient to a radical change of trajectory; (2) Entering the program can create a *jagged trajectory* that alternates between hope and renunciation and hence with intermittent positive effects); (3) Finally, experimental group participants only may experience a *split trajectory* because the program has a rebound effect, with a clear bifurcation between a pre-recovery and post-recovery course. The effects of the experimental program, as seen in the resulting trajectories, are strongest thus strongest in the case of the split trajectory. For the same person, program effects can change at a later stage, thereby producing a shift from a Type 1 trajectory to a Type 2 trajectory, or from a Type 2 trajectory to a Type 3 trajectory. The trajectories cannot be subsumed into one another. This is illustrated in the three examples (see Box 1: Shifts in trajectories produced by program effects).

***Shifts in Trajectories Produced* *by Program Effects***

**1.-Sabine**
**Cushioning Effect**
Type 1 Trajectory

Sabine was placed in foster care at age 4. She describes her early childhood as chaotic due to alcoholic parents. Her older sister died when she was 2. Later, her father came to get her at childcare. While still, as child, she was abused by an uncle. Despite a difficult childhood, she is proud of having been able to pursue her studies (“*I’m a literary type*”) and earn a degree as a veterinary assistant. She lived at her boyfriend’s. Then a man she says ruined her life beat her. She lost her job and home, and some of her people she met were negative. She spent five years homeless, most of the time living in a tent set up on a vacant lot, and then two years in prison. Her street life was basically surrounded by danger. She drank to cope and survived thanks to welfare (460 euros/month) and probably sex work. While on the streets, she gave birth to a baby girl who was taken away from her several days later. She wanted to keep the baby, but without housing or the presence of the child’s father, she preferred turning her over to foster care.

Sabine was 36 when she joined the *Un Chez Soi d’Abord* program. By this time, she had been taking monthly neuroleptic injections, under a judge’s order, for a year. She accepted housing after visiting five sites. She confided that this was the first time she had ever had a choice. At first, she saw housing as the end of a nightmare. She applied for housing entitlements, welfare, and disability entitlements, picked out furniture and decorated her apartment, got to know her neighbours, saw her friends from living rough at a bar she liked, joined in card games and *pétanque* (bocce) that the program organized. She stated explicitly that she wanted to change the course of her life, and she articulated realistic goals: find a part-time job to add to her disability entitlements, develop a normal lifestyle, hang out in cafés, get her teeth fixed, go to the hairdresser, ride the Ferris wheel, read. She wanted to find her father and get information about her daughter. On the health side, she changed from a treatment she did not like to oral medication that allowed her “*to find my ideas, again*”. She continued to drink a lot.

The first months were a “honeymoon”, even if Sabine took a while to reach out to the *Un Chez Soi d’Abord* team, as street life had taught her to look out for herself. For Sabine, the program was a means of moral and financial reintegration. “*Politically, that is all we need in life*”.

After several months, Sabine started pulling away from the team, lost weight, starting drinking a lot when by herself, stopped her medication, stopped paying rent and began to live behind closed shutters. She no longer took care either of herself or her space, ate badly, would scream at night, and could not sleep. She pulled herself together at times and tried detox despite the little effect it had on her. She tried to share pleasant moments with the team. From time to time, her pride about contributing to her rent was palpable. Two years after entering the program, her physical health deteriorated. If she seemed less affected by her mental health problems, she seemed tired out. Her attempts to locate her father, who never answered her letter, and her daughter, who was now a ward of the state, were in vain. Though she had struggled to overcome a difficult childhood (through studies, jobs, housing), her life was now filled with difficulty: diabetes, abdominal swelling, alcohol addiction. She lost hope:

*“When I was little, I was pretty, and healthy… I used to work. I had decided that work was my life, but my life was taken from me…. I hurt everywhere. I have fluid in my stomach, it’s an ascites [accumulation of fluid in the peritoneal cavity]”. “I’m going to die from my suffering … I’m diabetic, alcoholic, disabled… And everyone wants to hospitalize me. I’m sick of it.”*

“I’m not allowed to see my daughter … she needs to be adopted, to become a veterinary surgeon, to have a little money. I wish her happiness. If I take her back, she won’t be happy”.

**2-Fouad**
**Intermittent Positive Effects**
Type 2 Trajectory

Fouad, 30 years old, was exhausted when he joined the program. He took the first apartment he was shown. He had spent six years in the streets, without going to a drop-in center or getting housing. He had been hospitalized several times during his life. Before becoming homeless, he spent a year in a studio apartment, while being trained in carpentry in a social re-integration program. Before the studio, he lived with his mother, whom he used to fight with, as he did with his half-brothers and half-sisters. He mentioned his father only to say he had beaten his mother. When he got into housing, he sounded like a war veteran back from hell. Rather than actively envisioning a future, he saw it as a time when the effects of living on the streets would be erased. Fouad wanted “*to be in shape, physically and mentally*”. Housing now meant having the minimal support necessary to finding security and solace. The *Un Chez Soi d’Abord* team visit allowed him to have visitors and helped him with administrative procedures.

For several months, he tried to hide his drinking from the team. Drinking at home seemed to block out some of the voices he constantly struggled against. About a year into housing, he found himself in a critical state from heavy drinking and severe malnutrition. He was hospitalized, and it took several months of rehab to get him back on his feet. He experienced this situation as an impasse. But little by little he was able to find a routine with a precise schedule: day-hospital three days a week, shopping with the *Un Chez Soi d’Abord* team. Any change became a source of anxiety, including the very idea of having to move. He grudgingly acknowledged the effects of his medicine: “*It’s too strong*” but “*without it, I feel like someone is going to kill me. It’s like being possessed… But with the medicine, I don’t care about anything*”. At times, Fouad’s relationship with his voices structured his experience. Hope was intermittent. With the support of a peer worker provided by the team, he attended several self-help groups (Hearing Voices, Narcotics Anonymous, Alcoholics Anonymous) and, within two years of getting into housing, he began looking for alternatives to his dependency on alcohol and cannabis as means of quieting his voices. He sounded more optimistic and celebrated when only one of his five voices remained.

Fouad’s financial situation continues to be problematic after 24 months in the program, because he repaid a debt with the totality of his disability entitlements and still owes huge sum of money. Fouad managed to joke about his situation and hopes that his request for guardianship will be met. And the idea of going to a treatment center is motivated more by his need to eat than any desire of abstinence.

But the fear of losing the benefits of the program’s *accompagnement* is always present (“*I can’t do without them*”). His shame about never having done anything with his life blocks any attempt at reconciliation with his family, especially his mother: “*It’s bad to talk to someone like her, who is bipolar, and vice-versa given my state”.*

After two years, the social ties he has developed remain weak, but his network has expanded and diversified with more professionals, patients from the day hospital, a neighbor, other participants from the program and self-help groups. After two years into the program, he does not really feel he has control over his life. On the other hand, he is no longer in a survival mode and his everyday life contains moments of pleasure.

**3-Christian**
**Rebound Effect**
Type 3 Trajectory

Christian was 38 years old when he entered the *Un Chez Soi d’Abord* program. He had been residing in shelters for five years and had lived in a tent deep in the woods for seven years, where he suffered terribly from the cold and from hearing voices he qualified as terrifying. During this period of wandering, he lost all contact with his daughter, his mother (who experienced long-term psychiatric hospitalization for depression), and his father (who is retired). After a foot injury, he spent four months in a shelter for people with treatment needs. That is where a psychiatrist diagnosed him with schizophrenia, prescribed a neuroleptic and helped him apply for disability entitlements. He then spent six months in temporary housing, about which he has terrible memories (very bad hygiene conditions, a lowering of his entitlement benefits, bad company). He stayed in this facility until the *Un Chez Soi d’Abord* program was able to offer him housing, which despite its small size, gave him a sense of safety and solace.

Before falling into street life, Christian had been plumber for nine years, and lived in an apartment where, after his divorce, his daughter would come stay on weekends. He recalled that his voices worsened when his place was invaded by malevolent people who destroyed his apartment and his car (an important work tool). “*It started in 2004–2005. But I wasn’t in treatment. … I had no idea. I thought this was normal. At first, I thought we communicated through thought. But you know? It wasn’t normal.*”

Once Christian settled into his new home through the *Un Chez Soi d’Abord* program, he quickly reconnected with his daughter, mother and father. At the same time, Christian looked for work at the government employment agency, without success. At the beginning of the program, he received neuroleptic injections, but then he stopped for a while before starting again, thinking that they helped more than bothered him because of weight gain and fatigue. Neuroleptics silenced his voices, and at times he even felt cured. He talks about his schizophrenia, which a community mental health center monitors, but he does not identify with the “mentally ill”, claiming he is not that disabled.

Two years after moving into his new home, he gave up trying to find work.

His assessment of the financial pros and cons of living on disability entitlements as opposed to working as a plumber include the consideration that readapting to work might create distress that would be too risky for him.

Christian is relatively solitary. He avoids relationships that might disrupt the equilibrium he claims to have found. However, once in a while he sees his family and a friend. He checks in with the *Un Chez Soi d’Abord* team regularly, and feels positive about them. The team pays him weekly courtesy visits, and organize outdoor group activities such as fishing, going to the beach, or kayaking, which he enjoys. He says he doesn’t really need the team, but he “*has someone he can count on*”. He shows great interest in the program and gladly shares his thoughts on its efficiency, considering that the main obstacle to his success has been his drug and alcohol use (he smoked cannabis a lot for 15 years). He hasn’t completely met his aspirations: a normal life goes beyond just having an apartment. It means having a wife and kids.

## 6. Discussion

Overall, the results of the present study are consistent with those of previous studies that have attempted to define markers of recovery associated with Housing First-type programs. These include the return of “security and predictability in social and material environments”, the building of a routine, the reclamation of possible selves [23], a new learning process, a feeling of pride and security, and the development of meaningful activities [10,23].

This being said, several studies that have focused on the role of Housing First within the recovery process observe that certain participants remain demoralized by successive setbacks, isolated, vulnerable to trauma and addictions or involved in harmful social relations. These phenomena create obstacles to their engagement in positive transitions (from street to home, from home to community, and from the present to the future) [23], and in these cases, the program can at best be considered to be cushioning downward spirals.

By suggesting a close examination of the evolution of subjective relationships to objective life conditions, the data from this French study suggest that remedying the basic needs of people with a long history of life on the streets is necessary but not sufficient for their engagement in a recovery process. A parallel finding concerns the decisive role collaborative-type interventions on self-esteem and hope can play, despite the occasional persistence of major objective difficulties. In this sense, our observations also bring attention to the mechanisms at work among this minority of Housing First participants who engage in a path of recovery, against all expectations. Interestingly, this minority includes some members of the control group. Finally, this study explains how positive and negative so-called objective factors are equivocal as long as analyses do not include the subjectivity of the participant. Our analysis is in line with Davidson and colleagues [21], who conclude that every objective variable (housing, resources, work, social relations) should be related to the place it occupies within subjective experience, and hence to understanding whether or not it contributes to the recovery process.

A retrospective analysis thus seems crucial to pinpointing the subjective status of experience. For each individual interviewed, we can inquire about the meaning attributed to characteristics of past experiences. For example, is wandering reducible to violence? Family to support? Social relationships to drug user networks? This operation allows us to identify, for each individual, his or her own criteria for recovery.

The second analytical operation consists of thinking through the different dimensions of experience in the present, in order to pinpoint the virtuous or vicious circles which foster or impede the recovery process. For example, how does drug use or housing impact on the person’s social relationships, or mental health in daily activities?

The above-cited literature adequately describes the role played by Housing First in the recovery process. However, while these studies describe the associations between isolation and housing and between addictions and relationships, they rarely examine factors that undermine the possibility of projecting oneself in the future. Our analysis identified some of those obstacles, including administrative regulations and incarceration, increase in resources and the resurfacing of debts, somatic health and the return to work, rekindling of family ties and increase in resources, and access to care and mental health states. In other words, the French study shows that a synthesis and retrospective analysis of past experiences and a summary analysis of existing objective variables allow a refined analysis of personal factors of recovery.

Our qualitative study supports a notion of recovery emergent in the French context that goes beyond the remission of symptoms or stabilization of illness and resembles recovery as North Americans define it, that is as a process of coming to terms with the illness [44]. Within the framework of the *Un Chez Soi d’Abord* program, the recovery process is considered an institutional tool that allows professionals to help homeless people get off the streets and out of a life of wandering. The discourse of public policy actors who combat homelessness tends to classify good and bad trajectories according to the individual’s capacity to live in a home, confront their disorders, build social ties, or become employed. However proposing housing, care and leisure activities is insufficient for engaging in a recovery process. Their effectiveness as forms of support must also be shown. The qualitative research of the *Un Chez Soi d’Abord* program restitutes the singularity of the recovery process, which cannot be reduced to either a normalization of living conditions or symptom remission. Engagement in recovery involves a complex combination of factors that interact with one another. For France, the present study’s conclusions can contribute to understanding this as of yet scientifically unexplored process, one hardly promoted in public mental health policy.

## 7. Conclusions

This qualitative study from four French sites has documented the effects of the experimental program *Un Chez Soi d’Abord.* First, despite the late importation of the recovery notion into France, the findings are similar to those documented for Housing First programs in North America. Most often, this type of program for its participants is that of a refuge, and this generates an often-painful reflection on past life and hopefulness for future life. Although the meaning attributed to these changes varies according to the person’s previous experience, most individuals re-housed by this program go through a honeymoon period. Once the honeymoon is over, people search for a balance between protection and risk, attempt to solve dilemmas that access to the program reveals or cannot yet resolve, and confront practical and existential challenges.

Second, this study’s findings allow us to propose a classification of the program’s effects on life trajectories according to three modalities: (1) a *cushioning effect* on downward spiral trajectories preceding entry into the program, with a notable but insufficient effect of the program in the experimental group; (2) an *intermittently positive effect* on “jagged” trajectories that alternate periods of hope and renunciation, an effect only to be observed in the experimental group; (3) a *rebound effect*, found in both the experimental and the control group, in relation to a trajectory that was already spiraling downward before entry into the program.

Third, the qualitative analysis based on patient perspectives allows for a modified definition of recovery among people with psychiatric disorders who have experienced homelessness. The process of recovery can now be understood as the non-linear transformation of an experience (a relationship between objective life conditions and the subjective perception of these conditions), which reinforces protective support at the expense of risk elements. This tension between support and risk elements, in particular, contributes an original element to the understanding of recovery.

## Figures and Tables

**Table 1:** Domains of inquiry on homelessness and mental illness.

Initial Interviews—1–2 per Participant
Challenges, constraints and favorable aspects of participant’s life conditions
Relationship to the body, health, health behavior and coping
Aspirations and projections for the future
Relationship to home
Past and present relationships to services
Social relations (friends, family, urban acquaintances)
Places for sociocultural, leisure and pleasure
“Ontological sense”: security/insecurity
Social utility: social usefulness/uselessness
Well-being/malaise
Relationship (absent/present) to surrounding world
Beliefs, spiritual references, and relations to the religious.

**Table 2:** Formal interview guide.

Subsequent Interviews—2–4 per Participant
Trajectories of vulnerability
Helping relations and obstructive relations
Effectiveness of choices
Challenges to reconfiguration of identity
Challenges to experiential learning
Challenges to the appropriation of ability to act
Challenges to citizenship practices.

## Availability of Data and Material

The data sets generated and/or analyzed during the current study are not publicly available due to personal information but are available from the corresponding author upon reasonable request.

## Supplementary Materials

The following are available online at http://www.mdpi.com/1660-4601/15/3/520/s1, Table S1: Objective and subjective change criteria; Table S2: Scores on scale of factors affecting recovery and trajectory type.
