# Supplementary material for: Bread and Shoulders: Reversing the Downward Spiral, a Qualitative Analyses of the Effects of a Housing First-Type Program in France
Source: Int J Environ Res Public Health. 2018 Mar 14;15(3):520. doi: 10.3390/ijerph15030520 (PMC5877065; doi:10.3390/ijerph15030520)
Supplement: Supplementary file 1 [file ijerph-15-00520-s001.pdf]

Table S1: objective and subjective change criteria

12 « Objective » change criteria

Residential stability

1: no residential stability

2: weak residential stability

3: average residential stability (housing and shelter)

4: considerable residential stability (several successive housing episodes)

5: absolute residential stability (always in the same housing)

Reduction in consumption on substances/addiction

1: aggravation of consumption

2 : stability in consumption

3 : important consumption but less than before entering the program

4 : intermittent consumption

5 : no more consumption

Increase in resources

1: no resources

2: fewer resources than before

3: same amount of resources as before

4: somewhat more resources than before

5: many more resources than before

Eligibility for entitlements

1: loss of entitlements

2: same entitlements as before

3: access to entitlements

4: obtained several new entitlements

5 : all entitlements for which eligible

Social ties

1: break in social ties

2: no new social relationships outside those with professionals

3: at least one social relationship outside those with professionals

4: several new social relationships

5: increase in and diversification of social relationships

Family

1: no relationship to family

2: maintains a relationship with on family member

3: maintains a relationship with several family members

4: contact renewed with one family member

5: contact renewed with several family members

Sexuality/couple

1: no sexual relations or couple relationship

2: keeps a couple relationship

3: new relationship/lover

4: new couple relationship

5: Living in a couple

Work

1: no work

2: new training

3: intermittent work

4: return to part-time work

5: return to full-time work

Activities (outings-leisure-sports-cultural activities etc.)

1: no activities

2: fewer activities than before

3: same activities as before

4: a new activity

5: several new activities

Physical health

1: In worse health than before

2: In bad health like before

3: In good health like before

4: In better health than before

5: In much better health than before

Mental health

1: In worse health than before

2: In bad health like before

3: In good health like before

4: In better health than before

5: In much better health than before

Search for treatment, alternative resources

1: no treatment sought, like before

2: treatment seeking, like before

3: a new search for treatment

4: search for several types of treatment

5: search for treatment and alternative resources

6 « Subjective » change criteria

Hope, projection, goal-setting

1: no hope, like before

2: hope, like before, but no short-term goal-setting

3: more hope than before, but no short-term goal-setting

4: more hope, short-term goal setting, beginning to project future

5: more hope, beginning to project future, short- and medium-term goal setting

Reflexivity

1: No reflexivity, like before

2: beginnings of reflexivity

3: intermittent reflexivity

4: strong reflexivity (regarding self, others)

5: very strong reflexivity (regarding self, others, proximal environment and society)

Identity and social roles

1: Re-acquired a low-esteem identity, like before

2: re-acquired a lower-esteem identity

3: re-acquired a more positive identity

4: re-acquired an identity and new social role

5: required a positive identity and several new social roles

Ontological security

1: no sense of security, like before

2: weak sense of security, like before

3: medium sense of security, compared to before

4: sense of security about some aspects of life

5: complete sense of security

Symptom/disorder control

1: no control, like before

2: weak control, like before

3: occasional better control than before

4: good control of persistent symptoms

5: subjective absence of symptoms

Elaboration of strategies

1: no strategies, like before

2: some strategies, like before

3: a new strategy

4: several new strategies

5: several effective strategies

Table S2. Scores on scale of factors affecting recovery and trajectory type.

**Respondant**	**Summary calculation (12 objective variables; maximum score = 60). Percentages: actual summary score /maximum possible summary score.**	**Summary calculation (6 subjective variables: maximum score = 30) Percentages: actual summary score /maximum possible summary score.**	**Typology of trajectories***	
			Researcher 1	Researcher 2
Fouad	38 - 63%	18 – 60%	2	2
Sabine	33 - 55%	15 - 50%	1	1
Dylan	40 -66%	28– 93%	3	3
Christian	42 - 70%	25 – 83%	3	3
Tom	38 – 63%	22 – 73%	2	3
Dominique	43 – 71%	20- 66%	2	2
Hamed	32 - 53%	17 – 56%	2	2
Mouloud	31 – 51%	14 – 23%	2	2
Hervé	23 – 38%	14 – 23%	1	1
Raymond	35 – 58%	16 – 53%	2	2
Louis	42 – 70%	25 – 83%	3	3
Bernard	35 – 58%	22 – 73%	1	1
Elias	32 – 53%	20 – 66%	2	3

Legend : *Grey shading : experimental group; White shading: control group; *Type 1 trajectory, experimental group only: downward trajectory before entering the program with a* ***cushioning effect*** *on the downward course.*

Type 2 trajectory: « Jagged trajectory» with ***intermittent positive effect*** *on the trajectory. Type 3 trajectory: « Split» trajectory" with upward course and bifurcation, or* ***rebound effect***
